# The neural dynamics underlying the interpersonal effects of emotional expression on decision making

**DOI:** 10.1038/srep46651

**Published:** 2017-04-20

**Authors:** Xuhai Chen, Tingting Zheng, Lingzi Han, Yingchao Chang, Yangmei Luo

**Affiliations:** 1 Key Laboratory of Behavior and Cognitive Psychology in Shaanxi Province, School of Psychology, Shaanxi Normal University, Xi’an 710062, China; 2 Key Laboratory of Modern Teaching Technology, Ministry of Education, Shaanxi Normal University, Xi’an 710062, China

## Abstract

Although numerous studies explore the effects of emotion on decision-making, the existing research has mainly focused on the influence of intrapersonal emotions, leaving the influence of one person’s emotions on another’s decisions underestimated. To specify how interpersonal emotions shape decision-making and delineate the underlying neural dynamics involved, the present study examined brain responses to utilitarian feedback combined with angry or happy faces in competitive and cooperative contexts. Behavioral results showed that participants responded slower following losses than wins when competitors express happiness but responded faster following losses than wins when cooperators express anger. Importantly, angry faces in competitive context reversed the differentiation pattern of feedback-related negativity (FRN) between losses and wins and diminished the difference between losses and wins on both P300 and theta power, but only diminished the difference on FRN between losses and wins in cooperative context. However, when partner displays happiness, losses versus wins elicited larger FRN and theta power in competitive context but smaller P300 in both contexts. These results suggest that interpersonal emotions shape decisions during both automatic motivational salience valuation (FRN) and conscious cognitive appraisal (P300) stages of processing, in which different emotional expressions exert interpersonal influence through different routes.

How would you react to a competitor or a cooperator who displayed happy expressions when you lost money in a poker game? Would you trust a salesperson who smiles at you, or give money to a grumpy beggar muttering angry phrases? While such decisions are routinely made, little is known about how emotional expressions shape interpersonal decisions. Although contemporary scholars show increasing interest in identifying the effects of emotion on decision making, current efforts mainly focus on how individuals’ judgments and decisions are influenced by their own mood state (intrapersonal effect)^1^,^2^, leaving the influence of one person’s emotions on the other’s decisions and behavior (interpersonal effect)^3^,^4^,^5^ less understood. Therefore, here we sought to address how interpersonal emotional expressions shape decision making and elucidate the underlying neural dynamics thereof, using the high temporal resolution of electroencephalography (EEG).

So far, some studies have investigated the interpersonal effect of emotional expressions. For instance, it has been shown that people are more likely to cooperate with smiling partners^6^. Further, they appear to overweight the positive outcomes associated with happy faces and underweight the positive outcomes associated with angry faces^7^,^8^. Moreover, people made fewer risky decisions when a friend expressed anxiety via a video link^9^. They made more advantageous choices in a congruent emotional context, but more disadvantageous choices in an incongruent emotional context^10^. Additionally, emotion displays within interpersonal contexts can impact negotiation^11^,^12^,^13^, dispute resolution^14^, reactions to ultimatums^15^, trust^16^, and prosocial behaviors^17^,^18^.

Despite the fact that an interpersonal effect of emotional expressions has repeatedly been demonstrated, the mechanisms underlying this are not fully understood. One line of study implicates emotion contagion in the effect. Emotion contagion occurs when people automatically mimic each other’s facial expressions and bodily postures. Interoceptive feedback from these reactions then produces corresponding emotions^19^,^20^. Additionally, some theorists assume that the effects of exposure to expressions do not necessarily involve direct mimicry. Rather, they reflect implicitly perceived meaning of the expressions through embodied processes^21^,^22^. A contrasting line of study suggests that emotional expressions shape other people’s responses through reverse appraisal, in which people draw on their knowledge of appraisal–emotion associations to make inferences about other person’s appraisals^23^,^24^. Integrating existing theory and research, the emotions as social information (EASI) model developed by Van Kleef^4^,^5^,^25^ distinguishes two ways (affective reactions and inferential processes) in which emotion exerts interpersonal influence. The model holds that the depth of information processing and perceived appropriateness of the other person’s emotion determines whether inferential or affective processes play a part.

While previous research depicts the interpersonal effect of emotional expression in decision making and external moderators thereof^3^, it does not probe the neural dynamics underlying such an effect. This is of importance since depiction of the neural dynamics can further elucidate the underlying mechanisms of the interpersonal effect of emotional expressions. One possible answer is that other’s emotional expressions affect decision making through modulating both the early coarse detection of motivational significance and the late elaborated appraisal of the motivational significance of the feedbacks, analogous to the modulation of utilitarian feedback on decision making^26^,^27^,^28^,^29^,^30^.

The quick detection of the motivational or valence significance of a feedback is associated with feedback-related negativity (FRN), a negative component peaking at fronto-central electrodes roughly 250–300 ms after presentation of relevant feedback information^26^,^31^. Usually, the FRN was reported to be larger after negative feedback (e.g. monetary losses or angry expressions) on task performance compared to positive feedback (e.g. monetary gains or happy expressions)^26^,^32^,^33^,^34^,^35^,^36^,^37^. Although the FRN has traditionally been thought to be specifically elicited by bad action feedback^26^, it has been proposed that this ERP effect might represent a reward positivity, a positive-going ERP component that is specifically elicited by good action feedback^38^,^39^. Conversely, the more elaborated and conscious appraisal of the motivational significance of an outcome is linked with P300, or described as a feedback-related P300, a positive deflection occurring between 350–600 ms after feedback presentation with a parietal distribution^27^,^28^,^29^,^30^,^35^,^40^,^41^,^42^,^43^,^44^. The P300 has been attributed to working memory updating after unexpected events and the evaluation of task relevance^45^ and in the case of feedback processing reflects a later, elaborated and conscious appraisal of the motivational significance of an outcome^29^,^41^. It was found that the P300 is sensitive to various aspects of outcome evaluation, including the magnitude of reward^35^,^41^, expectancy toward the outcome^43^,^46^, and the valence of the outcome^27^,^29^,^35^,^41^,^42^,^46^. In addition to the two time-domain averaged signatures (FRN and P300), frontal midline theta power is also said to be sensitive to the feedback processing^47^,^48^,^49^,^50^,^51^. Although it is thought to be responsive to the same underlying processes as the FRN, the theta activity in/over the medial frontal cortex, including anterior cingulate cortex (ACC), is believed to reflect the process of cognitive control more closely, given that it provides non-phase locked oscillatory power^48^,^49^,^50^,^51^,^52^. Modulations in theta-band activity have shown to be sensitive to feedback manipulations in various aspects, including unexpectedness^47^,^53^,^54^, risk-relatedness^55^ as well as feedback-related error information^48^,^51^.

Although some studies have examined the neural dynamics underlying emotional feedback processing^32^,^56^, most of these studies have focused exclusively on the emotional cues alone when participant act as a passive spectator. Given that people are embedded in social interaction that shapes their brains throughout lifetime, it is necessary to adopt ecologically valid studies including interacting participants^57^. Additionally, effective real-life decision making requires the integration of many sources of information, especially utilitarian cues and cues from the social context^7^,^16^. Thus, it is ecologically unfit to explore the interpersonal effects of emotional expressions alone^32^,^58^, without a consideration of the interaction with other factors, such as utilitarian cues.

For these reasons, the current study aims to specify the neural dynamics of interpersonal effects of emotional expressions in decision making, after taking account of the interaction between utiliarian and emotional cues. We carried out an experiment with a modified version of Gehring and Willoughby’s gambling task^26^, in which participants chose between two monetary options in each trial. After choice decision, they received a feedback that combined utilitarian cues and emotional cues orthogonally. The utilitarian cues indicated whether the choice resulted in win or loss, while the emotional cues indicated whether one’s partner was happy or angry with a given outcome. Moreover, in order to test whether the interpersonal effect was context dependent or not, cooperative and competitive contexts were established.

We focused on the FRN, feedback-related P300, as well as theta synchronization to seek converging evidences, because of their strong link with feedback processing^26^,^35^,^47^. However, the inherent ERP components linking with emotional expression processing seemed to be inevitable since the feedback in the current study consists of emotional and utilitarian cues. Therefore, the well-known components in the processing of emotional displays, particularly facial expressions^59^,^60^ were also analyzed. Those components include occipito-temporal N170^59^,^61^,^62^,^63^ and fronto-central vertex positive potential (VPP)^59^,^63^,^64^,^65^ reflecting attentional capture by emotion, occipito-temporal early posterior negativity (EPN)^61^,^66^,^67^ and fronto-central N2^59^,^68^,^69^,^70^ indexing affective discrimination, parietal P300 reflecting sustained attention and elaborative categorization processes^59^,^60^,^61^,^71^,^72^, and fronto-central slow positive wave (SPW) linking with response selection and decision^59^,^66^. Relevant to the current study, the difference between happy and angry expressions was examined. In this regard, the previous studies showed larger EPN^59^, larger P3b and SPW^59^ for angry faces than for happy faces, but smaller N2 for angry faces than for happy faces when faces were presented in a trust-varied context^73^. For conciseness, following previous studies^32^,^58^, the current study merged the analysis on FRN and N2, and the analysis on feedback-related P300 and P3b, given that the FRN is a special instance of the N2 component^74^,^75^,^76^,^77^ and the feedback-related P300 belongs to P300 family^40^,^41^,^42^,^43^.

We hope to replicate the interpersonal effect of emotional expressions^3^,^4^,^5^, which might be indexed by a modulation of emotional cues on both behavioral and brain responses associated with feedback. Given that expressing anger may be perceived as inappropriate in East Asian cultures^11^, we predicted that angry faces upon one’s wins might be perceived as unfavorable independent of contexts through affective reactions^5^,^25^, and thus shrink the difference on the FRN, feedback-P300 between losses and wins^30^,^39^. Conversely, on the basis of that the appropriateness of expressing happiness varies as function of context^25^, we hypothesized that happy displays upon one’s loss might be perceived as unfavorable in competitive context but favorable in cooperative context through inferential processes^5^,^25^, and consequently demonstrate different modulation effect on the FRN, feedback-P300. Moreover, since the theta power is thought to be responsive to the same underlying processes as the FRN^48^, we hope that the theta power could provide converging evidence as FRN.

## Method

### Participants

Forty right-handed university students (20 female) ages 18–27 years were recruited to participate in this experiment. The mean age of the sample was 20.5 years old (SD = 2.14). They were randomly assigned to either competitive or cooperative games, with 20 participants (10 female) in each. All participants reported normal auditory and normal or corrected-to-normal visual acuity, and were free of neurological or psychiatric problems. They were emotionally stable, as evidenced by the significant below-threshold (0) scoring in neuroticism assessment [M = −4.64; S.E. = 5.73; *t*(36) = −4.86, *P* < 0.001] of the shortened Chinese version of the Costa and McCrae NEO-FFI^78^. Four participants (2 women) were excluded from analysis due to excessive EEG artifacts in the recordings. The experimental procedure adhered to the Declaration of Helsinki and was approved by the approved by the Ethical Committee of Shaanxi Normal University. All participants gave informed consent prior to the study.

### Procedure

Upon entry into the lab, the participant was introduced to a confederate of the same gender who would perform as a partner in a gambling game through a computer network. Participants’ facial expressions (happy, angry, and neutral) were recorded using a Canon EOS 600D and used as feedback stimuli in the experiment. Unbeknownst to the participant, the facial expression of the confederate was prerecorded and validated in advance. A base payment of ¥40 was given to participants to use for gambling. Participants were given an additional reward or punishment based on their performance, resulting in earnings ranging from ¥30 to ¥50. Then they were randomly assigned to either a cooperative or competitive context. In the cooperative context, the participant was told that the obtained outcomes of the participant and the confederate would be added together and be equally distributed between them. In contrast, participants in the competitive context were told that the confederate was an opponent to their interest. That is to say, a loss by the participant means a win by his or her opponent in the same amount, and vice versa.

**Figure 1:**
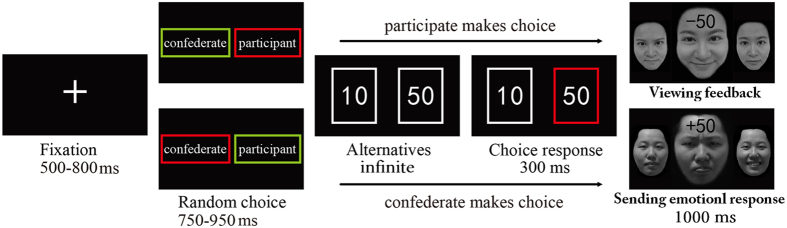
Schematic diagram of an experimental trial in the interpersonal gambling task. After a fixation, the computer selected the one who performs the current round of gambling by flickering the name of the participant and confederate (the one whose name is circled with a red square would be the performer and the one whose name is circled with a green square would be the observer). Then, they both viewed the alternatives and the performer chose one of the squares by pressing the corresponding button. After the current choice was presented for 300 ms, the observer would send his or her emotional responses to the performer by pressing one of the emotional expressions, and the performer would receive feedback combined with utilitarian (“+” means win and “−” means loss) and emotional cues (happy/angry/neutral). The three facial expressions were presented to illustrate the experimental design here and only the middle selection expression was presented to the participant in the experiment.

The classic Gehring and Willoughby gambling task^26^ was adapted for the interpersonal gambling game in this study. Figure 1 shows a schematic diagram of a trial in this task. Specifically, after a fixation period, the participants were told that the computer would randomly select the performer and the observer in the current round of gambling first. The person selected as the performer would view two squares, each of which contained the numeral 10 or 50 (cents), and make a choice by pressing the corresponding button as soon as possible. After the choice was presented for 300 ms, the feedback was presented for 1000 ms, in which a “+” on the forehead of the partner’s face indicated that the participant won the points whereas a “−” indicated that the participant lost the points. The happy or angry expression of the partner indicated that he or she was happy or angry with the current outcome. At the same time, the person selected as the observer would observe the performer’s choice and send his or her emotional response to the partner. Unbeknownst to the participant, the choices and affective responses of the confederate were predetermined. Trials with neutral expressions were included as fillers to make the game more realistic but were not included in the data analysis. The whole experiment consisted of 534 trials divided into 8 blocks. In each block the participant performed two thirds of the trials, while the confederate performed the remaining third, (which was not used in the data analysis).

### EEG recording

EEG measurements were recorded at 64 scalp sites using tin electrodes mounted in an elastic cap (Brain Product, Munich, Germany) according to the modified expanded 10–20 system, each referenced online to FCZ. Vertical electrooculogram (EOG) was recorded supra-orbitally and infra-orbitally from the right eye. The horizontal EOG was recorded as the left versus right orbital rim. The EEG and EOG measurements were amplified using a 0.05–100 Hz bandpass and continuously digitized at 1000 Hz for offline analysis. The impedance of all electrodes was kept less than 5 kΩ.

### Data analysis

#### Preprocessing

**Figure 2:**
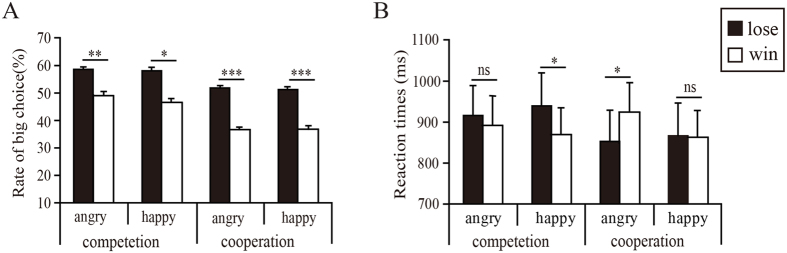
Mean (**A**) rates of risky choice after wins and losses, and (**B**) the corresponding reaction times, as a function of conditions. Error bars indicate standard error.

The “10” is defined as the low-risk option (small potential win or loss) while the “50” is defined as the high-risk option (large potential win or loss). The tendency to choose the high-risk option indicates a preference for risk-seeking strategy. This preference was measured as the “risk ratio” by dividing the number of risk-seeking choices by the total number of choices. Following previous studies^26^,^28^,^35^, we analyzed the preceding outcome on risky behavior in the current trial. Thus, the risk ratio of the second trial during consecutive trials and the corresponding reaction times (RTs beyond three standard deviations were excluded in RT calculation) were calculated as the dependent variable (see Fig. 2).

EEG data was preprocessed using EEGLAB^79^, an open source toolbox running on the MATLAB platform. First, the data were down sampled at 250 Hz, high pass filtered at 0.5 Hz, and re-referenced offline to bilateral mastoid electrodes. The data were segmented into epochs around the presentation of outcome feedback stimuli (1000 to 2000 ms post stimulus). The epoched data were baseline corrected using 200 ms before the onset of the feedback. EEG epochs with large artifacts (exceeding ± 100 μV) were removed, and channels with poor signal quality were interpolated using EEGLAB toolbox. Trials contaminated by eye blinks and other artifacts were corrected using an independent component analysis algorithm^79^. There were 58.45 ± 3.82, 58.55 ± 4.30, 58.55 ± 2.70, and 58.95 ± 3.75 artifact-free trials obtained for the lose-angry, lose-happy, win-angry, and win-happy conditions in the cooperative context, and 58.20 ± 4.80, 58.40 ± 3.86, 59.35 ± 2.81, and 58.95 ± 4.58 artifact-free trials obtained for these four conditions in the competitive context. Note that the magnitude (10 vs. 50) of the outcome was collapsed for conciseness, since our primary purpose was to investigate the interaction of utilitarian and affective cues in outcome evaluation.

**Figure 3:**
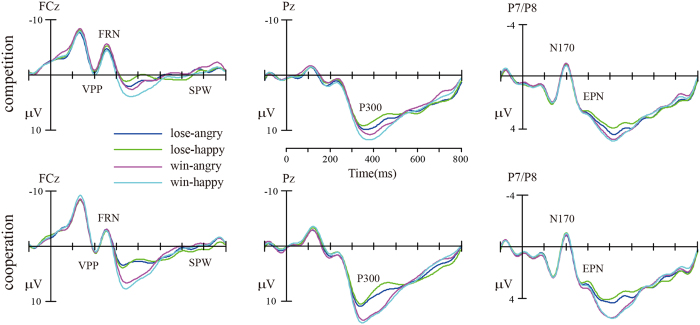
Group-averaged ERP voltage waveforms over fronto-central (FCz), parietal (Pz), and occipito-temporal (P7/P8) representative electrodes.

**Figure 4:**
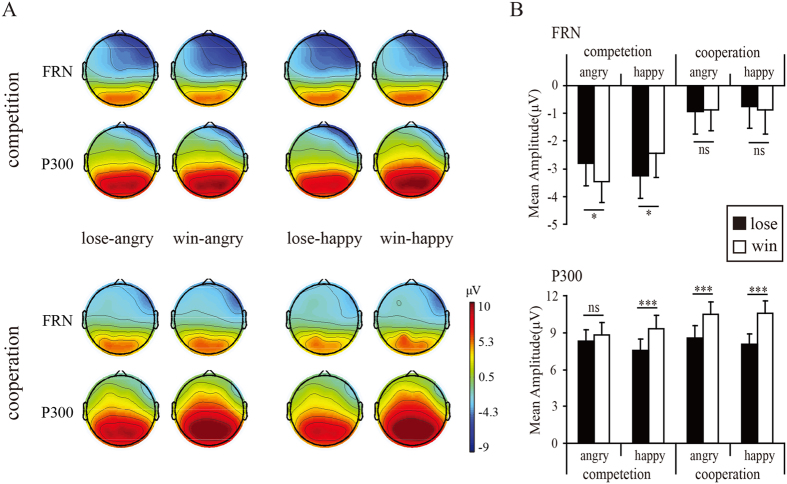
(**A**) Scalp topography (top view shown) for FRN and P300 during the selected time window, and (**B**) bar graphs of the mean amplitudes as function of conditions.

For the event-related potential (ERP) analysis, the artifact free data was re-segmented to 1000 ms epochs time-locked to the onset of feedback, starting 200 ms prior to the onset of feedback. The data were then low-pass filtered at 30 Hz. The extracted average waveforms for each participant and condition were used to calculate grand-average waveforms. For statistical analyses, three different clusters of scalp sites were formed to evaluate the components peaking in different regions^59^: fronto-central (electrodes: F1, Fz, F2, FC1, FCz, FC2, FCz, C1, Cz, C2), occipito-temporal (left: TP7, P7, PO7, and right: TP8, P8, PO8), and parietal (P1, Pz, P2, PO3, POz, PO4). Then, based on the inspection of visual peaks for the current data, the mean amplitude in the fronto-central cluster was calculated for the interval between 180 and 220 ms for the VPP, between 220 and 280 ms for the N2/FRN, and between 600 and 800 ms for the SPW. Similarly, the mean activity in the occipito-temporal cluster was calculated for the interval between 180 and 220 ms for the N170 and between 280 and 320 ms for the EPN. Finally, to assess the feedback P300, the mean activity at the parietal cluster was calculated between 300 and 500 ms (see Figs 3 and 4).

**Figure 5:**
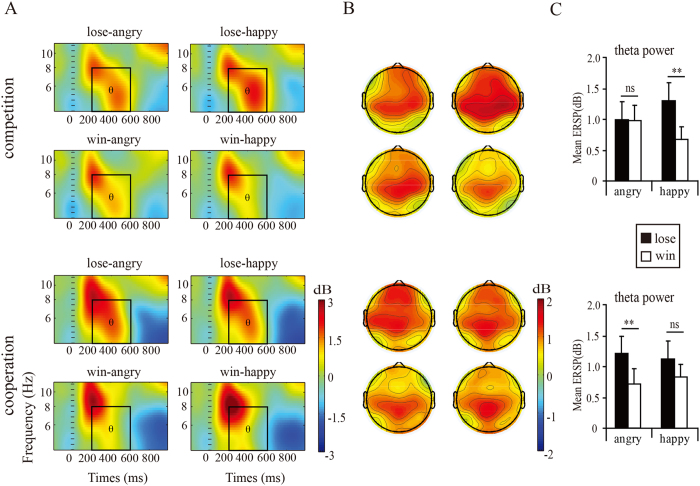
(**A**) Group-averaged ERSP over CZ, (**B**) scalp topography (top view shown) for theta power, and (**B**) bar graphs of the mean power as function of conditions.

For the event related spectral perturbation (ERSP) analysis, the artifact free data was applied to wavelet decomposition using EEGLAB12.0.1.0b. Changes in event-related spectral power response (in dB) were computed by the ERSP index (1)^79^,


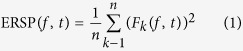


where for n trials, *F*_*k*_ *(f,t)* is the spectral estimate of trial *k* at frequency *f* and time *t*. Power values were normalized with respect to a 200 ms pre-splicing point baseline and transformed into a decibel scale (10_log10 of the signal). ERSPs were averaged over trials for each condition and transformed into time-frequency plots. For conciseness, only the data of interest (4 to 15 Hz over −200 to 1000 ms) is presented. Based on the permutation test implemented in the statcond function of EEGLAB and previous studies addressing theta synchronization underlying performance monitoring processes^47^,^48^,^49^,^50^,^51^, ERSP in the theta band (4–8 Hz) during 200–600 ms over FZ, FCZ, and CZ were averaged for statistical analysis, (see Fig. 5 for ERSP).

### Statistical analysis

We first confirmed that the ratio of choice was different from the chance level by comparing the actual choice ratio with the chance level (50%). Then we entered the behavioral data and EEG data into repeated measures ANOVAs, with the *outcome valence* (loss vs. win) and *emotion* (happy vs. angry) as within-subject factors and *context* (cooperative vs. competitive) as a between-subject factor. The degrees of freedom of the F-ratio were corrected per the Greenhouse–Geisser method, and multiple comparisons were Bonferroni adjusted in all these analyses. The effect sizes are shown as partial eta squared (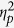).

## Results

The participants selected more high-risk options after losses (0.55 ± 0.02) than after wins (0.43 ± 0.03), [*F*(1,34) = 32.49, *p* < 0.001, 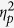 = 0.49], and selected more high-risk options in the competitive context (0.54 ± 0.04) than in the cooperative context (0.43 ± 0.04), [*F*(1,34) = 5.28, *p* = 0.02, 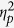 = 0.13], which is significantly different from the chance level (0.50). However, no significant main effect of *emotion* or any interaction involving *emotion* was observed [*Fs = *(1, 34) < 0.76, *p* > 0.39], (see Fig. 2A).

The analysis on reaction times showed significant interactions of *outcome valence* × *context* [*F* (1,34) = 4.86, *p* = 0.034, 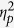 = 0.113], and *emotion* × *outcome valence* [*F*(1,38) = 8.59, *p* = 0.006, 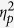 = 0.184]. To probe these interactions, planned comparisons between wins and losses under all emotional cues and each context were carried out. As can be seen in Fig. 4B, the reaction times were significantly longer following losses (939 ± 77 ms) than following wins (887 ± 65 ms, *p* = 0.029) when accompanied by a happy expression in the competitive context. In the condition that combined the cooperative context with an angry expression, reaction times were significantly shorter after losses (853 ± 72 ms) than after wins (925 ± 71 ms, *p* < 0.017).

The analysis of FRN amplitudes showed a significant interaction of *emotion* × *outcome* [*F*(1,34) = 4.25, *p* = 0.047, 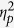 = 0.11], and a significant interaction of *emotion* × *outcome valence* × *context,* [*F*(1,34) = 7.29, *p* = 0.01, 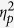 = 0.18]. Simple effects analysis indicated that, in the competitive context, the losses (−2.81 ± 0.81 μV) elicited smaller FRN amplitudes than the wins (−3.45 ± 0.75 μV, *p* = 0.027) when accompanied by angry facial expressions, but elicited larger FRN amplitudes (−3.27 ± 0.80 μV) than wins (−2.43 ± 0.88 μV, *p* = 0.019) when accompanied by happy facial expressions. In contrast, in the cooperative context, the FRN amplitudes for losses and wins were not significantly different regardless of affective cues, as shown in Fig.4C. The other way of simple effects analysis only yielded that competitor’s angry expressions (−3.45 ± 0.75 μV) elicited larger N2 amplitudes than happy expressions (−2.43 ± 0.88 μV, *p* = 0.006) did, when the participants won the game.

The analysis of P300 amplitudes showed a significant main effect of *outcome valence* [*F*(1,34) = 55.90, *p* < 0.001, 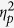 = 0.62], a significant interaction of *emotion* × *outcome valence* [*F*(1,34) = 6.12, *p* = 0.019, 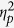 = 0.15], and a significant interaction of *outcome valence* × *context,* [*F*(1,34) = 5.42, *p* = 0.026, 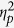 = 0.14]. Simple effects analysis yielded that, in the competitive context, the wins (9.34 ± 1.07 μV) elicited larger P300 amplitudes than the losses (7.59 ± 0.87 μV, *p* < 0.001) when accompanied with happy facial expressions, while the P300 differences between wins (8.85 ± 0.99 μV) and losses (8. 30 ± 0.96 μV, *p* = 0.13) were diminished when accompanied by angry expressions. In contrast, under the cooperative context, the wins elicited larger P300 than losses when accompanied by both angry (10.49 ± 0.99 μV vs. 8.60 ± 0.96 μV, *p* < 0.001) and happy (10.55 ± 1.07 μV vs. 8.05 ± 0.87 μV, *p* < 0.001) expressions.

The analysis of theta activity showed a significant main effect of *outcome valence* [*F*(1,34) = 10.34, *p* = 0.003, 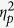 = 0.23], and a significant interaction of *affective* × *outcome valence* × *context,* [*F*(1,38) = 5.34, *p* = 0.027, 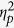 = 0.13]. Simple effects analysis yielded that, under the competitive context, the losses (1.30 ± 0.30 dB) induced a larger theta power than wins (0.67 ± 0.20 dB, *p* < 0.01) when accompanied by happy faces, but the theta power for losses (1. 00 ± 0.29 dB) and wins (0.98 ± 0.26 dB, *p* = 0.91) were not significantly different when accompanied by angry faces. In contrast, under the cooperative context, the losses (1.21 ± 0.28 dB) elicited larger theta power than wins(0.71 ± 0.26 dB, *p* = 0.01) when accompanied by both angry faces, but no significant difference between losses (1.12 ± 0.30 dB) and wins (0.83 ± 0.20 dB, *p* = 0.19) when combined with happy faces.

The analysis of VPP, N170, EPN and SPW amplitudes showed a significant main effect of *outcome valence* for EPN [*F*(1,34) = 7.58, *p* = 0.009, 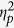 = 0.18], with the smaller amplitudes for losses (3.11 ± 0.34 μV) than for wins (3.41 ± 0.36 μV), as well as a significant main effect of *outcome valence* for SPW [*F*(1,34) = 4.34, *p* = 0.045, 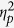 = 0.11], with the more negative amplitudes for wins (−0.49 ± 0.47 μV) than for losses (−0.03 ± 0.45 μV). However, no interaction involving *outcome valence* or *context* was observed [*Fs* (1,34) < 3.84, *p* > 0.05] for all these components. Critically, no significant main effect of *emotion* [*Fs* (1,34) < 2.80, *p* = 0.103] and interactions involving *emotion* [*Fs* (1,34) < 3.84, *p* > 0.05] were observed, suggesting that emotional expressions did not differ regardless of utilitarian cues and contexts.

## Discussion

This study examined behavioral and brain responses to utilitarian feedback combined with emotional expressions in both competitive and cooperative contexts to explore the neural dynamics underlying how emotional expressions shape interpersonal decisions. In line with previous studies 26,28,35, participants selected more high-risk options following losses relative to wins, replicating the classic influence of utilitarian cues on an individual’s decision making. Moreover, reaction times were slower following loss relative to win trials when a competitor displayed happiness at the outcome, suggesting that happiness in a competitive context may be taken as unfavorable, probably schadenfreude^80^. Additionally, participants made slower responses following wins relative to loss when their cooperator displayed anger at the outcome, consistent with the previous study showing that anger in a cooperative context is perceived as inappropriate^5^.

More importantly, angry faces in the competitive context reversed the differentiation pattern of FRN, and diminished the difference between losses and wins on both feedback P300 and theta band power, partly consistent with our prediction. FRN^32^,^33^,^34^,^35^,^36^,^37^ and theta activity^47^,^48^,^49^,^50^,^51^are thought to be associated with unfavorable relative to favorable feedback, while P300 is larger for favorable relative to unfavorable feedback^27^,^29^,^35^,^40^,^41^,^42^,^46^. In this regard, it is understandable that competitor’s angry faces were taken as negative feedback, which consequently reduced the positive-going deflection elicited by wins, and lessened the difference between losses and wins on P300 amplitudes. However, the reversed differentiation pattern on FRN cannot be explained with this theory. In contrast, based on the new proposal that FRN represents reward positivity specifically elicited by favorable feedback^44^, we speculate that the negative valence embedded in angry expressions counteracted positive valence linking with wins and consequently decreased the positive deflection elicited by wins. Moreover, as there is evidence suggesting a similar functional implication shared by FRN and theta oscillation during outcome evaluation^48^, we thought the same mechanism could explain the simultaneous reduction in theta power between losses and wins.

Even though the new interpretation of FRN can account for the current results, the bigger FRN for wins relative to losses is still a little bit elusive. We speculate the comparison between the ERPs associated with two emotional expressions following wins and losses separately may help to explain this. It has been proposed that the negative activity in the 200–300 ms time window, referred to as the FRN or N2 component, has consistently been observed to reflect valence difference^69^,^70^,^74^,^75^,^76^,^77^. A competitor’s happy faces following one’s losses are of more negative valence than angry faces, and thus elicited larger negative deflection. Conversely, a competitor’s angry faces following one’s wins elicited larger negative deflection than happy faces did because they are of more negative valence. Although the difference in N2 amplitude between angry and happy expressions was not frequently reported^59^,^60^, the current finding is in line with the study reporting a smaller N2 for angry faces than happy faces in an interpersonal context^73^. This suggests that facial expressions processing might be modulated by utilitarian context.

When a competitor displayed happiness, losses were associated with larger FRN and theta power, but smaller P300. In this regard, happy expression in competitive context seems to have no profound influence on the valence of the utilitarian feedback. This might result from that the happy expressions following losses were taken as negative feedback (schadenfreude^80^), but were taken as positive following wins, at least not so bad as angry expressions. The interpretation of happy expressions can also be validated by behavioral response that reaction times were slower following losses than following wins when a competitor displayed happiness at the outcome.

In the cooperative context, there were no significant differences in FRN amplitudes between losses and win during both emotional displays. This might result from the cancellation of valence between utilitarian and emotional feedbacks: while cooperator’s angry faces upon one’s wins were taken as unfavorable feedback, cooperator’s happy faces upon one’s losses were taken as favorable feedback, probably a consolation to the loss. This phenomenon was also supported by the slower responses for wins relative to loss when their cooperator displayed anger. However, the similar modulation of emotional expressions did not extend to P300 range, as losses elicited smaller P300 relative to wins regardless of partner’s emotional feedback. This may indicate that emotional feedback do not have so much influence the elaborated appraisal of the motivational significance of the outcome^27^,^28^,^29^,^30^,^40^,^41^,^42^,^43^ in the cooperative context.

Together, the current findings demonstrated that interpersonal emotions modulate the feedback processing during different time range, and such influence varied depending on contexts. The modulation of other’s expressions on feedback processing is consistent with previous studies showing that emotional information biases decision-making^7^,^8^,^9^,^10^. Moreover, the modulation of emotional cues is also in agreement with findings that the brain activities associated with feedback processing are modulated by social cues such as interpersonal relationship^27^, diffusion of responsibility^46^, social comparison^81^,^82^, and social conformity^83^. Different from the previous studies^7^,^8^,^9^,^10^, emotional cues in the current study were online emotional responses of a partner in a realistic gambling game, ensuring that the modulation of emotional cues can be taken as an interpersonal effect^3^,^4^,^5^. Thus, the current study provides the first neurophysiological evidence for the interpersonal effect of emotion expressions in decision making^3^,^4^,^5^. This complements previous studies on the interpersonal effect of emotional expressions in various behavioral performances, such as negotiation^11^,^12^,^13^, trust^16^, and prosocial behaviors^17^,^18^,

Obviously, the influence of other’s emotional feedbacks varied depending on emotionality and context. As proposed by the EASI model^4^,^5^,^25^, emotional expressions influence one’s decisions by eliciting reciprocal affective reactions in observers or eliciting inferential processing through which observers glean information to arrive at decisions. Judging from the modulation effect on brain indicators, angry expressions in both competitive and cooperative contexts were all perceived as a negative feedback. We speculate that angry expression exerts its influence through reciprocal affective reactions, because of its low appropriateness regardless of contexts. This speculation is reasonable given that angry display is not appreciated in the Chinese culture^11^,^25^. However, the appropriateness of a happy expression depends on the context, and is more likely to prompt thorough information processing. Thus, the dissociation of the influence of happy expressions implicates an inferential processing when happy faces come into play. These findings are in line with previous findings that joy after mutual cooperation leads to more cooperation, whereas joy after exploitation results in less cooperation^23^,^84^. Together, supporting the EASI model^4^,^5^,^25^, the current findings suggest that different emotional expressions might exert their influence through different routes, and perceived appropriateness of the particular emotional displays determine the relative impact of affective reaction versus inferential processes.

The modulation of emotional expressions on different brain indicators also sheds light on the neural dynamics of the interpersonal effects of emotional expressions. Given that FRN is associated with automatic motivational evaluation of outcomes^32^,^33^,^34^,^35^,^36^,^37^,^38^, and P300 reflected an elaborate and conscious appraisal of the motivational significance of an outcome^27^,^29^,^40^,^41^,^42^, the current findings suggest that emotional expressions shape interpersonal decisions in both the early stage of automatic monitoring of motivational salience and the late stage of cognitive appraisal processing. The modulation of emotional expressions was also observed on frontal midline theta power. Based on the implication that the medial frontal theta activity is a neural signature of cognitive control and characterizes a general process of controlling attention^51^,^52^, we speculate that interpersonal emotional expressions shape decisions by modifying attention assignment.

The other thing worth noting is that we did not observe significant interaction between emotional and utilitarian cues on the components associated with facial expressions. The null effect of emotion on early components (N170, VPP and EPN) suggested that both happy and angry expressions share same level of attentional allocation and emotional arousal in the current experimental context, in line with the previous studies reporting no significant difference in early components between angry and happy expressions^59^,^60^. Counter to the previous studies, we did not observed significant difference in SPW, a component believed to reflect categorization and response selection^59^,^66^. This might be due the current study did not require participants make explicit decision regarding facial expressions.

Despite the contributions of this study, some limitations should be noted. For the sake of brevity, we did not examine the potential effects of different magnitudes of monetary rewards. Though findings have not been completely consistent, some research suggests that magnitude of monetary reward may influence modulation of emotion expression on utilitarian feedback. For instance, magnitude of reward has been reported to affect FRN^33^. For this reason, future research should directly test for an interaction between reward magnitude and interpersonal emotion. Another limitation of the present study is that we adapted the Gehring and Willoughby gambling task^26^ so that it required an interpersonal social decision. However, in realistic settings, interpersonal emotion expressions might be transient and implicit to individuals. Thus, to more accurately depict the neural dynamics associated with the interpersonal effects of emotion on decision making, future studies should investigate these factors in naturalistic settings. Further, studies presenting emotional expressions unconsciously might provide more direct evidence of the effects found here.

## Conclusion

The present study examined both behavioral and brain responses to utilitarian feedback combined with emotional expressions in both competitive and cooperative contexts to specify the neural dynamics underlying the interpersonal effects of emotional expressions. As predicted, interpersonal emotional expressions affect one’s response latency, FRN, P300, and theta power change associated with outcome evaluation, and such modulation varies depending on context. These findings indicate that emotional expressions shape interpersonal decisions during both automatic motivational salience valuation and the conscious cognitive appraisal stages of processing, in which happy expressions mainly elicit inferential processing while angry expressions elicit affective reactions in exerting interpersonal influence.

## Additional Information

**How to cite this article**: Chen, X. *et al*. The neural dynamics underlying the interpersonal effects of emotional expression on decision making. *Sci. Rep.* **7**, 46651; doi: 10.1038/srep46651 (2017).

**Publisher's note:** Springer Nature remains neutral with regard to jurisdictional claims in published maps and institutional affiliations.
